# Brain white matter integrity and cortisol in older men: the Lothian Birth Cohort 1936^☆^

**DOI:** 10.1016/j.neurobiolaging.2014.06.022

**Published:** 2015-01

**Authors:** Simon R. Cox, Mark E. Bastin, Karen J. Ferguson, Susana Muñoz Maniega, Sarah E. MacPherson, Ian J. Deary, Joanna M. Wardlaw, Alasdair M.J. MacLullich

**Affiliations:** a Centre for Cognitive Ageing and Cognitive Epidemiology, University of Edinburgh, Edinburgh, UK; b Brain Research Imaging Centre, Division of Neuroimaging Sciences, University of Edinburgh, Edinburgh, UK; c Department of Psychology, University of Edinburgh, Edinburgh, UK; d Scottish Imaging Network: A Platform for Scientific Excellence (SINAPSE) Collaboration, Edinburgh, UK; e Edinburgh Delirium Research Group, Geriatric Medicine Unit, University of Edinburgh, Edinburgh, UK; f Endocrinology Unit, University of Edinburgh, Edinburgh, UK

**Keywords:** Cortisol, Glucocorticoid, White matter, Aging, Brain structure, Tractography

## Abstract

Elevated glucocorticoid (GC) levels are hypothesized to be deleterious to some brain regions, including white matter (WM). Older age is accompanied by increased between-participant variation in GC levels, yet relationships between WM integrity and cortisol levels in older humans are underexplored. Moreover, it is unclear whether GC-WM associations might be general or pathway specific. We analyzed relationships between salivary cortisol (diurnal and reactive) and general measures of brain WM hyperintensity (WMH) volume, fractional anisotropy (g_FA_), and mean diffusivity (g_MD_) in 90 males, aged 73 years. Significant associations were predominantly found between cortisol measures and WMHs and g_MD_ but not g_FA_. Higher cortisol at the start of a mild cognitive stressor was associated with higher WMH and g_MD_. Higher cortisol at the end was associated with greater WMHs. A constant or increasing cortisol level during cognitive testing was associated with lower g_MD_. Tract-specific bases of these associations implicated anterior thalamic radiation, uncinate, and arcuate and inferior longitudinal fasciculi. The cognitive sequelae of these relationships, above other covariates, are a priority for future study.

## Highlights

• We correlated salivary cortisol and brain white matter (WM) measures in older males.
• Cortisol was measured diurnally and in reaction to a cognitive challenge.
• Diffusion tensor magnetic resonance imaging (fractional anisotropy and mean diffusivity) and total hyperintensity volume measured WM integrity.
• WM-cortisol relations were found for mean diffusivity and hyperintensity volume but not fractional anisotropy.
• Higher cortisol in response to cognitive stressor denoted lower WM integrity.

## 1 Introduction

Glucocorticoids (GCs; cortisol in humans) are produced via activation of the hypothalamic-pituitary-adrenal (HPA) axis in response to stress. They also exhibit a diurnal rhythm, with high levels in the morning which decline during the day. Sustained chronic exposure to high GCs through stress or exogenous administration has deleterious effects on brain structure and function in animals (McEwen and Gianaros, 2010). A similar effect is observed in Cushing disease in humans, a disease whose hallmark is chronically elevated cortisol (Patil et al., 2007). In some animals and humans, aging is accompanied by flatter diurnal slopes, lower GC levels on waking (Heaney et al., 2010), and increased GC levels in reaction to stress (Otte et al., 2005). A flatter cortisol slope is considered to represent HPA axis dysregulation, whereby the negative feedback loop fails to reduce cortisol levels either in line with the normal diurnal pattern, or following cortisol elevation in response to an environmental catalyst (Herman et al., 2003). Aging and GC levels are hypothesized to interact in complex ways dependent on cell type; some such interactions may affect the brain adversely and contribute to cognitive decline (Landfield et al., 2007; Sapolsky et al., 1986). However, research examining the cerebral correlates of these complex age-GC interactions has mainly focused on cortical and subcortical loci that comprise the GC regulatory network. Little empirical research has examined how GCs might relate to the integrity of white matter (WM) tracts, some of which connect these regions. Moreover, studies investigating the neurostructural correlates of GCs in older age tend not to consider both diurnal and reactive measures together.

HPA axis activity is modulated by a complex network of regions including the hippocampus, amygdala, and prefrontal cortex (Ulrich-Lai and Herman, 2009). These regions appear particularly sensitive to the detrimental effects of excess GCs. Prolonged exposure to repeated instances of restraint stress or exogenous steroids in animals results in reduced synaptic and dendritic complexity in the hippocampus and medial prefrontal cortex (reviewed in McEwen and Gianaros, 2010). In older humans, reduced anterior cingulate volume (MacLullich et al., 2006) and thinner lateral frontal cortex (Kremen et al., 2010) are associated with markers of HPA axis dysregulation. However, models of the brain network(s) that influence HPA axis regulation (Dedovic et al., 2009) have underplayed the role of WM integrity, and studies of GC effects on WM are scarce. WM is essential in facilitating efficient information transfer between cerebral regions (Filley, 2010) and plays a central role in the body's response to environmental and homeostatic challenge (Ulrich-Lai and Herman, 2009). Poorer integrity of this connectivity via any mechanism could therefore also lead to impaired operation of GC regulation and impaired cognition, just as it would for relevant cortical and subcortical sites.

The theoretical importance of WM in HPA axis control in aging and disease is supported when considering that elevated GC levels impair axonal sprouting in response to insult. Following experimental lesions in rodents, WM repair by axonal sprouting was significantly reduced in a dose-dependent manner by exposure to a glucocorticoid receptor agonist compared with controls (Scheff and Cotman, 1982; Scheff and DeKosky, 1983). Increased exposure to GCs pre-lesion led to less axonal sprouting than when GCs were administered only post-lesion (Scheff and Dekosky, 1989). Moreover, GCs and stress inhibit the proliferation of astrocytes and oligodendrocytes in animal models (Rajkowska and Miguel-Hidalgo, 2007). Higher GC levels might result in a reduced repair response to accumulated damage via any route and affect the maintenance of axonal myelination. This could also lead to further impairment of HPA axis regulation as information transfer becomes compromised, impairing GC regulation and further impairing reparative responses to subsequent WM insult (Sapolsky et al., 1986).

Measures of the magnitude of water molecule diffusion (mean diffusivity; MD) and its directional coherence (fractional anisotropy; FA) obtained from diffusion tensor magnetic resonance imaging (DT-MRI) allow an estimate of WM microstructure. More tightly packed fiber bundles characteristically exhibit higher FA and lower MD, and comparison of these DT-MRI biomarkers and postmortem histopathology suggests that FA and MD are reliable indices of reduced axonal integrity, demyelination, and accumulation of extracellular fluid in frontotemporal dementia (Larsson et al., 2004), multiple sclerosis (Schmierer et al., 2007), and older age (Kochunov et al., 2007; Sullivan and Pfefferbaum, 2007). Using DT-MRI, tractography can measure the 3-dimensional structure and integrity of major axonal fiber bundles, providing generally good concordance with WM fibers identified postmortem (Catani and Thiebaut de Schotten, 2008; Klein et al., 2010; Wakana et al., 2004). Additionally, WMHs are a common feature of the aging brain, observable on T_2_ and fluid-attenuated inversion recovery (FLAIR)-weighted MRI (Longstreth et al., 2000; Schmidt et al., 2011). The total WM hyperintensity (WMH) load can therefore be quantitatively assessed by measuring the total volume of WMHs and offers a neuroradiologically complementary metric of WM integrity to the diffusion-based measures discussed previously. Using both approaches, we are able to estimate WM integrity both at the level of major connective fiber pathways and at the level of aggregate brain-wide volume of visible age-related WM deterioration.

Few studies have examined the association between indices of HPA axis activity and brain WM integrity measured using DT-MRI. Increased MD, indicating lower WM structural integrity, measured in callosal, uncinate, and cingulum bundles was associated with higher observational rating of behavioral stress reactivity but not directly with 12 hours urinary cortisol in rhesus monkeys (Willette et al., 2012). In humans, one study reported an inverse relationship between evening cortisol levels and a measure of periventricular WM integrity using a region of interest approach in small healthy sample (n = 23, mean age = 43.9, SD = 11.6 years; Macritchie et al., 2013). Given that the hippocampus, amygdala, and prefrontal cortex have been centrally implicated in supra-HPA axis regulation (Dedovic et al., 2009; Ulrich-Lai and Herman, 2009) findings that associate specific fiber integrity with indices of HPA axis dysregulation are partially commensurate with the underlying neuroanatomy. For example, the uncinate fasciculus arises lateral to the hippocampus and amygdala, connecting entorhinal cortex with prefrontal cortex. The cingulum bundle runs from the parahippocampal gyrus and uncus of the temporal lobe and runs superior to the corpus callosum into the frontal lobes. Similarly, the anterior thalamic radiation (ATR) is a major efferent pathway between the prefrontal cortex (PFC) and thalamus; the anterior thalamic nucleus receives afferents from the hippocampus and anterior projections extend predominantly to the cingulate cortex and lateral PFC (Catani and Thiebaut de Schotten, 2008; Nolte and Angevine, 1995). Consequently, the ATR, uncinate, and cingulum bundles are plausible candidates to examine the hypothesis of tissue specific relationships with cortisol levels among older humans.

In summary, elevated GCs and flatter slopes in aging are possible contributors to brain and cognitive aging, and this might be partly because of the effects on WM integrity. General WM integrity is significantly associated with processing speed, which in turn is strongly associated with higher general cognitive ability in older age (Penke et al., 2012). Thus, disruption of information transfer by WM damage in older age could partially explain impairments to both cognitive ability and GC regulation. However, it is unclear whether the hypothesized interactions between aging and cortisol are differentially expressed among the brain's various long-range WM tracts, and also across various measures of the complex cortisol profile, whose characteristics do not show a unidirectional change with increasing age. The limited animal and human data are relatively underpowered and have not explicitly addressed cortisol-WM relationships in aging nor have they examined the contribution of both reactive and diurnal GC levels. We therefore measured the total volume of WMHs, the water diffusion characteristics of 7 major long-range WM tracts and both diurnal and reactive cortisol levels in a group of 90 healthy older men. We hypothesized that higher reactive cortisol levels and flatter slopes (between sampling points on the same occasion) would correlate with lower WM integrity, as indicated by higher WMH volume, lower FA, and higher MD.

## 2 Methods

### 2.1 Participants

Ninety male participants from the Lothian Birth Cohort 1936 (LBC1936) participated in this study. Details of this cohort have been described previously (Deary et al., 2007, 2012). All participants who scored <11 on the Hospital Anxiety and Depression Scale (HADS) (Zigmond and Snaith, 1983) were not taking antidepressant or GC medication, made no self-report of stroke, had no history of serious neurologic event such as large brain infarcts, meningiomas, frontal or temporal cysts, or extensive siderosis, frontal infarcts (all MRI scans were examined by a consultant neuroradiologist, Joanna M. Wardlaw) were free from a diagnosis of neurodegenerative disorders, and scored 24 or above on the Mini-Mental State Examination (MMSE; Folstein et al., 1975). Participants were of mean age 73.30 (SD 0.37) years at MRI scanning. Salivary cortisol sampling took place just over 1 year after MRI acquisition (mean 431.42 days, SD 103.62). Written informed consent was obtained from each participant and the study was conducted in compliance with departmental guidelines on participant testing and the Declaration of Helsinki. Ethical approval was gained from NHS Lothian Research Ethics Committee (07/MRE10/58) and the Philosophy, Psychology, and Language Sciences Research Ethics Committee at the University of Edinburgh. The testing appointment took place in the Department of Psychology, University of Edinburgh (a distinct site from the Wellcome Trust Clinical Research Facility, Western General Hospital, Edinburgh where previous testing appointments had been conducted) and participants were informed they would be undergoing a battery of cognitive tests with which they were unfamiliar. The appointment format and tests used are detailed in Supplementary Material.

### 2.2 Cortisol

As a measure of reactive cortisol production, saliva was collected using Salivette devices (Sarstedt, Numbrecht, Germany) before and after a morning cognitive testing appointment (approximately 90 minutes apart). These collection points will be referred to as start and end. The appointment was expected to act as a mild stressor with higher levels at the start of testing because it took place in an unfamiliar, unpredictable, and uncontrolled environment, and participants were anticipating cognitive ability tests which they had not seen before (Lupien et al., 2007). On a separate weekday, saliva was collected at home on waking and at 10 PM to characterize the diurnal cortisol profile. These collection points will be referred to as waking and evening. Diurnal slope and reactive slope were calculated by subtracting the earlier measure (waking or start) from the later measure (evening or end). Thus, a negative value denotes a decreasing slope. Cortisol units are nmol/L.

Following collection, salivettes were stored at −80 °C. Assays were carried out by Dresden LabService GmbH (Dresden, Germany) in accordance with a Material Transfer Agreement. Intra-assay variation was <20% at the lower limit of quantitation and <15% above that. Correlation coefficients for regression lines were >0.99, consistent with typical international standards.

### 2.3 MRI acquisition

Full details of the brain MRI protocol, including figures illustrating the range of images acquired, are available in Wardlaw et al. (2011). Briefly, participants were scanned using a GE Signa LX 1.5T MRI clinical scanner with a self-shielding gradient set with maximum gradient strength of 33 mT/m, and 8-channel phased-array head coil. Image acquisition comprised whole brain T2-, T2*- and FLAIR-weighted axial scans, a high-resolution 3D T1-weighted volume sequence acquired in the coronal plane (voxel dimensions 1 × 1 × 1.3 mm), from which neuroradiological assessment was made. Single-shot, spin-echo, echo-planar, diffusion-weighted volumes (b = 1000 s/mm^2^) were acquired in 64 noncollinear directions, along with 7 T2-weighted images (b = 0 s/mm^2^). Seventy-two contiguous axial slices of 2 mm thickness were acquired with a field of view of 256 × 256 mm and matrix size of 128 × 128, giving a resolution of 2 × 2 × 2 mm^3^. Repetition and echo times were 16.5 seconds and 95.5 ms, respectively. Total image acquisition took approximately 70 minutes.

### 2.4 White matter lesion measurement

Quantitative measurement of WMHs was performed using MCMxxVI (Hernández et al., 2010) which segments and records WMH volume in voxels (converted to mm^3^ for statistical analysis). WMHs did not significantly correlate with intracranial volume (*r* [89] = 0.16, *p* = 0.13) and correcting for intracranial volume had no significant effect on the magnitude of reported associations (Supplementary Table 1). WMHs were identified as punctate or diffuse areas in the WM or basal ganglia in the vertebral hemispheres or brain stem that were hyperintense with respect to normal-appearing white matter and gray matter on T_2_-weighted and FLAIR images. They could show some hypointensities on T_1_-weighted MRI but were not as hypointense as the cerebrospinal fluid signal. The initial segmentation was visually inspected in each case and manually corrected for any false positive tissue structures (e.g., artefacts) or any incorrectly omitted WMHs.

### 2.5 Tractography

Data were initially preprocessed to extract the brain, remove bulk participant motion and eddy current-induced distortions, and estimate water diffusion tensor parameters using FSL tools (FMRIB, Oxford UK; http://www.fmrib.ox.ac.uk/). Brain connectivity data were created using the BEDPOSTX/ProbTrackX tractography algorithm with a 2-fiber model and 5000 streamlines to reconstruct tracts of interest. Twelve major long range WM tracts thought to be related to cognitive functioning (Penke et al., 2012; genu and splenium of corpus callosum, and bilateral ATR, cingulum bundles, uncinate, arcuate, and inferior longitudinal fasciculi [ILF]) were identified from these connectivity data using probabilistic neighborhood tractography as implemented in the TractoR package for fiber tracking and analysis (http://www.tractor-mri.org.uk/; Clayden et al., 2011). This technique optimizes the choice of seed point for tractography by estimating the best matching tract from a series of candidate tracts generated from a neighborhood of voxels (typically 7 × 7 × 7) placed around a voxel transferred from standard to native space against a reference tract that was derived from a digital human white-matter atlas (Bastin et al., 2010). An example of an MD image and the candidate tracts are shown in Supplementary Fig. 1. The topological tract model was also used to reject any false positive connections, thereby significantly improving tract segmentation (Clayden et al., 2011). For each participant, the seed point that produced the best match tract to the reference for each of the 12 pathways was determined, with the resulting tractography mask applied to each participant's MD and FA volumes. Tract-averaged values were calculated from these masks and used in all subsequent analyses. To ensure that the segmented tracts were anatomically plausible representations of the fasciculi of interest, a researcher (Susana Munoz Maniega) visually inspected all masks blind to the other study variables and excluded tracts with aberrant or truncated pathways.

### 2.6 Statistical analysis

Before analysis, all data were examined for extreme values without knowledge of their relationships with other variables of interest. All data were examined for normality and the presence of outlying values (±3 SD). Outlying FA and MD values were examined in the first instance and removed where they clearly represented measurement error or partial volume effects. If the measurement accurately represented the brain region or tract of interest, these were winsorized to preserve relevant data but minimize the disproportionate effect that outliers have on parametric statistics. All (12) outlying cortisol data points were removed from further analysis (rather than winsorized) because there is a weaker correspondence between salivary and serum cortisol with increasing concentrations (Hellhammer et al., 2009). Log-transformed cortisol measures for evening, start and end, and square-root transform of WMH volume rectified skewed distributions. Pearson product-moment correlation coefficient (*r*) is reported throughout. To check whether the data transformation steps disproportionately complicated the interpretability of findings, nonparametric (Spearman rank) correlations were also conducted for inclusion in the Supplementary Material. To investigate the possible neuroendocrine effect of the novel testing environment, we compared raw start cortisol with the value that would be predicted by each participant's linearly modeled diurnal rhythm (start–predicted value), accounting for individual differences in sampling times. Observed start levels greater or equal to the value predicted by a participant's diurnal model at the time of start sampling is a conservative estimate of the effect of attending the appointment, given that the diurnal rhythm is highly nonlinear, showing a sharp decline from 30 minutes postwaking (e.g., Evans et al., 2011; Heaney et al., 2010).

Tract-averaged FA and MD values for the segmented tracts were all positively intercorrelated with similar magnitudes as previously described for the whole LBC1936 cohort from which current participants were drawn (range for FA measures *r* = 0.15–0.58, mean = 0.51; range for MD measures *r* = 0.32–0.69, mean = 0.39; Penke et al., 2012) apart from splenium MD which generally correlated less strongly with all other MD measures (range *r* = 0.07–0.23, mean = 0.11). We therefore used 2 principal components analyses to derive a general white matter measure each for FA (g_FA_) and MD (g_MD_). After entering all tracts into the analysis, we extracted the first unrotated component in each analysis. g_FA_ and g_MD_ were positively loaded by all tracts and explained 50.48% and 51.39% of the shared variance, respectively. This data reduction enabled us to examine relationships between cortisol at different sampling points and general brain WM tract integrity as indicated by 3 biomarkers (WMHs, g_FA_, and g_MD_). We also correlated MD and FA of each of the 7 individual tracts (genu and splenium of corpus callosum, and averaged bilateral ATR, cingulum bundles, uncinate, arcuate, and ILF) with cortisol levels to identify any potential tract-specific effects that general indices of integrity may have partially obscured.

## 3 Results

### 3.1 Descriptive statistics

Descriptive statistics for salivary cortisol levels for diurnal (waking, evening, and diurnal slope) and at the cognitive testing appointment (start, end, and reactive slope) are shown in Table 1. Participants had a mean Mini-Mental State Examination score of 28.54 of 30 (SD = 1.52), a mean HADS-A score of 3.97 (SD = 2.71), and HADS-D of 2.74 (SD = 2.33). Mean cortisol sampling times (self-report for home samples) were: start: 10:30 AM (SD = 32 minutes, range 9:15–11:55 AM); end: 12:06 PM (SD = 137 minutes, range 10:30–1:35 PM); waking: 7:52 AM (SD = 1 hour, range 5–10 AM); evening: 10:02 PM (SD = 21 minutes, range 8–11 PM). In general, waking levels were higher than evening levels (t = 17.67, df = 100.47, *p* < 0.001), and start levels were higher than end levels (t = 3.97, df = 145.80, *p* < 0.001). The average slope for participants was negative for both diurnal and reactive measures. For diurnal slope, all were negative but 15% of participants showed a relatively flat slope of <10 nmol/L. For reactive slope, 26% had positive slopes, 31% showed a change between 0 and −5 nmol/L, and the rest showed negative slopes (between −5 and −20.12 nmol/L). Supplementary Fig.2 and 3 illustrate participants' diurnal and reactive cortisol profiles, respectively. Over one-third of participants (34.15%) exhibited start levels higher than that would be expected at the start sampling time in a linear model of each participant's diurnal slope. Moreover, the mean of −4.60 (SD = 11.18 nmol/L) indicated that most (52%) of the participants' start levels were either higher or <5 nmol/L lower than a straight line between waking and evening would predict. Table 1 also shows the FA and MD characteristics of the major long-range WM tracts.

### 3.2 Cortisol levels and general white matter integrity

Correlations of salivary cortisol measures with WMH volume, g_FA_, and g_MD_ are reported in Table 2. No correlations were significant between any cortisol measure and g_FA._ WMH volume was significantly correlated with start (*r* [85] = 0.24, *p* = 0.026) and end (*r* [88] = 0.21, *p* = 0.048) cortisol levels. Supplementary Fig. 4 shows scatterplots of these relationships. gMD correlated positively with start cortisol levels (*r* [78] = 0.25, *p* = 0.031), suggesting that higher cortisol levels at the start of cognitive testing were indicative of lower WM integrity (greater g_MD_). In contrast, g_MD_ correlated negatively with the reactive slope (*r* [78] = −0.39, *p* = 0.0005). This indicates that the more positive the reactive slope, the higher the degree of WM integrity as assessed by lower g_MD_. There was a trend toward a positive relationship between evening cortisol and g_MD_ (*r* [78] = 0.23, *p* = 0.053). Nonparametric tests of these associations (Supplementary Table 2) suggest that data transformation for parametric analysis did not disproportionately affect interpretability of the previously reported results.

That WMH was significantly correlated with both start and end levels but not with reactive slope suggested a possible interaction. We used partial correlations to explore this, controlling the WMH-reactive relationship for start and end levels separately. Partialling out start levels attenuated this relationship somewhat (*r* [82] = 0.05, *p* = 0.627), whereas controlling for end levels qualitatively increased its magnitude (*r* [82] = −0.18, *p* = 0.093). Although nonsignificant, this could suggest that, for 2 people with the same end levels, the one with a more positive reactive slope may have lower WMH volume.

### 3.3 Cortisol levels and tract-specific integrity

**Fig. 1:**
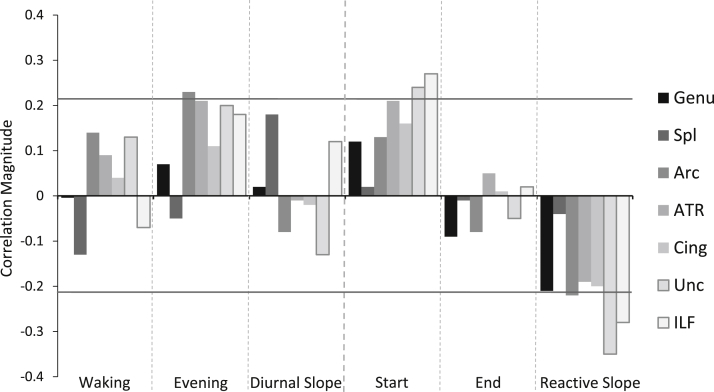
Tract-specific correlations between white matter mean diffusivity and cortisol levels. Horizontal lines give an indication of significance threshold, Evening, Start and End cortisol measures are log transformed. Abbreviations: Arc, arcuate fasciculus; ATR, anterior thalamic radiation; Cing: cingulum bundle, ILF, inferior longitudinal fasciculus; Spl, splenium of the corpus callosum; Unc, uncinate fasciculus.

Correlations between cortisol measures and individual tract integrity are reported in Table 3. Correlation magnitudes are also shown in Fig. 1 to illustrate the general and tract-specific relationships between MD and cortisol. Consistent with our analysis of g_MD_, tract-specific associations were mainly found with evening, start, and reactive slope cortisol measures. MD values in the arcuate, ATR, uncinate, and ILF showed directionally consistent relationships with evening (range *r* = 0.18–0.23), start (range *r* = 0.13–0.27), and reactive slope (range *r* = −0.19 to −0.35) cortisol. Although these correlations did not reach significance in all cases, Fig. 1 demonstrates the consistency of correlation magnitudes and directions among these tracts and within cortisol sampling points.

Relationships between cortisol and g_FA_ were generally nonsignificant. There was only 1 significant FA-cortisol association, between the splenium of corpus callosum and diurnal slope (*r* [82] = −0.22, *p* = 0.05). Just as with the g_FA_ and g_MD_ analysis, there were no significant correlations between tract-specific integrity (FA or MD) and either waking or end cortisol levels.

## 4 Discussion

In this study, we combined diurnal and reactive cortisol sampling with indices of WM integrity to investigate their complex hypothesized relationships in a sample of older humans. These data suggest that elevated cortisol levels in the evening or at the start or end of a mild cognitive stressor may relate to generally poorer WM integrity in older age. However, our data also indicate that increasing or stable cortisol during this cognitive stressor was indicative of generally lower WM diffusivity (which may be indicative of better integrity). When we explored the tract-specific nature of these effects, they were most consistently driven by cortisol's relationship with MD of the arcuate, ATR, uncinate, and ILF across these cortisol sampling points.

We did not correct our analyses for multiple comparisons because FA and MD measures are, in themselves, not independent, and there is a large degree of shared variance between different tracts (e.g., Penke et al., 2012). It is therefore likely that conventional type I error correction would be overly conservative in this situation because each new comparison would not represent an entirely independent opportunity for type I error to arise (Nyholt, 2001; Ott, 1999). In addition, correlation magnitudes were of a consistent direction within sampling points–particularly for evening, start, and reactive slope measures–and the same tracts showed consistent correlations with cortisol across sampling points; neither of which can be easily explained by chance. Finally, comparisons of cortisol with FA and MD measures were conducted simultaneously, and the resultant split between null and significant findings between these 2 WM measures is not consonant with a patterning of significant findings because of chance. Although these data require replication in a larger sample to further examine their validity, the nature and context of these findings merits further discussion.

The finding that elevated start cortisol is linked to greater g_MD_ and WMHs, and end cortisol with greater WMHs (indicative of lower WM integrity) is in line with the hypothesis outlined previously, whereby elevated GCs in aging humans may be associated with brain aging. However, a flatter or more positive reactive slope (increasing or maintaining levels during cognitive testing) was associated with lower tract g_MD_. One interpretation of these data could be that superior integrity confers the ability to (appropriately) maintain cortisol output during this type of stressor, overriding the inhibitory signals of the HPA axis negative feedback loop.

Our hypothesis was founded on the assumption that high levels of cortisol at both reactive time points and a flatter slope might indicate elevated exposure to cortisol in response to stressful events and impaired negative feedback once acclimatized, with resultant lower WM integrity. Thus, higher start cortisol would signify a response to a novel situation in a novel location and anticipation of a mild cognitive stressor, whereas lower end levels might reflect the efficiency of the negative feedback loop in reducing these levels with acclimatization to the testing environment (Lupien et al., 2007). However, higher end levels and flat and/or positive reactive slope might also represent the continuing appropriate excitation of HPA axis activity in the face of continuing cognitive demand, particularly among a group of healthy individuals who were motivated to participate in a longitudinal study. Thus, a failure to respond during a time of perceived need could also be considered pathologic, and previous work provides some support for this concept. Blunted cortisol response to a cognitive stressor (the Trier Social Stress Test; Kirschbaum et al., 1993) was identified in younger individuals suffering from chronically high work stress (Bellingrath and Kudielka, 2008). Reduced cortisol response to a cognitive task was also reported among individuals with damage to frontal brain regions (Lueken et al., 2009). Moreover, higher cortisol at the end of a functional MRI memory paradigm was positively correlated with task-related frontal lobe BOLD response and memory performance in a young but not old group (Kukolja et al., 2008). This could indicate a biological response to cognitive demand that continues into older age but which may cease to be of cognitive benefit. The relationship between cortisol levels and cognitive scores will be addressed in future studies.

In the absence of a direct precedent, the validity of tract-specific relationships with cortisol in older humans can be examined by considering whether they are biologically plausible and in the context of related studies. The uncinate fasciculus arises lateral to the hippocampus and amygdala, connecting entorhinal cortex with prefrontal cortex (Catani and Thiebaut de Schotten, 2008; Nolte and Angevine, 1995). Similarly, the ATR is a major efferent pathway between the PFC and thalamus; the anterior thalamic nucleus receives afferent connections from the hippocampus and anterior projections extend predominantly to the cingulate cortex and lateral PFC. Hippocampus, amygdala, PFC, and cingulate cortex have each been implicated in GC regulation (Dedovic et al., 2009; Ulrich-Lai and Herman, 2009). Increased MD in this tract is associated with increased behavioral reactivity to stressful situations in rhesus monkeys (Willette et al., 2012). Both the connectivity and the functional role of the arcuate fasciculus are under continuing research (Bernal and Ardila, 2009), but it has not yet been implicated in endocrine regulation. Likewise, the ILF showed the same general pattern of greater MD with evening and start, and lower MD with reactive slope cortisol measures. It connects temporal and occipital lobes (Bergman and Afifi, 2005; Catani and Thiebaut de Schotten, 2008), and neither lobe has been a significant focus of attention in the neuroendocrine literature, aside from the medial temporal lobe. However, it is very interesting to note that the tracts most strongly linked with cortisol in this study are predominantly frontal or temporal fibers. Such fibers generally take longer to reach maturation (i.e., are late myelinating) during development, show delayed declines in middle age (Lebel et al., 2012), and those with the most protracted development may be particularly susceptible to decline in older age (Bartzokis, 2004; Gunning-Dixon et al., 2009). Combined with the known involvement of frontal and temporal brain regions in cortisol regulation (Dedovic et al., 2009; Ulrich-Lai and Herman, 2009), the present data suggest the possibility that late-myelinating tracts may be more susceptible to the adverse effects of elevated GCs. This possibility could be explored in future studies.

The absence of significant findings with tract FA is conspicuous when compared with the MD results; we report that cortisol levels are generally related to the magnitude of water molecule diffusion within certain tracts but not with its directional coherence. This dissociation suggests interpretative caution because FA and MD values tend to both correlate with postmortem measures of myelination in multiple sclerosis, a disorder known to affect WM structure (Schmierer et al., 2007) although another study reported that MD, but not FA was shown to change over time in the WM of progressive multiple sclerosis patients over 2.4 years (Agosta et al., 2007). Nevertheless, this FA and/or MD split has precedent in relation to reactive stress. A study of cortisol and DT-MRI biomarkers in the rhesus monkey reported a significant interaction between behavioral stress reactivity and urinary cortisol in MD, but not FA, of limbic tracts (Willette et al., 2012). Although the biological significance of MD and FA is under debate (Hasan, 2006), this pattern of findings could reflect slight tissue damage which is sufficient to increase extracellular water but not to significantly alter the fiber coherence and consequent directional diffusion of water molecules (MacLullich et al., 2009). MacLullich et al. (2009) reported significantly greater MD but not FA of normal appearing WM in association with higher blood pressure among males aged 71–76 years. They contend that hypertension could increase blood-brain-barrier (BBB) permeability, resulting in an increase in extracellular water. GCs are also intimately involved in BBB permeability (Lubrich et al., 2000). Although an acute (medical and/or therapeutic) increase in GC levels decreases BBB permeability (Förster et al., 2005), the effects of chronic modest GC exposure on the aging BBB are unknown, warranting further investigation.

An additional reason to interpret these data with caution is as follows. Although elevated start cortisol is indicative of higher WMH volume and higher g_MD_, the fact that the correlations of these 2 WM measures with cortisol are not entirely consistent for end and reactive slope (Table 2) makes it difficult to conclude simply that reactive cortisol levels might be causally linked to poorer WM integrity. As previously mentioned, WMH and MD measure fundamentally different WM characteristics; the former represents the total volume of observable focal lesions across the brain's WM, whereas the latter represents average water molecule diffusion only across a series of 7 long-range fasciculi. Thus, it is possible that these data simply reflect differential involvement of supra-HPA axis neuroregulatory networks in different phases of the reactive response, some of which do not involve WM in the 7 long-range tracts that comprise g_MD_ (i.e., end cortisol) and some of which are particularly pertinent to them (i.e., the reactive slope). Nevertheless, this pattern of correlations could equally suggest that cortisol exposure is not the causal mechanism, and future work should examine whether and to what degree appropriate covariates previously associated with WM decline such as hypertension or blood pressure (Hernández et al., 2013) attenuate the associations reported here.

This study uniquely combined diurnal and reactive cortisol measures with detailed measurement of WMHs and water diffusion parameters for major long-range WM pathways. It was conducted in a healthy aging sample with a narrow range of ages, which eliminated most of the potentially confounding effect of variations in age. Nevertheless, there are several limitations. Previous research suggests that waking and evening sampling points are sufficient to capture diurnal cortisol rhythms, but we used only a single sampling occasion whereas sampling on at least 2 days has been recommended (Kraemer et al., 2006). In particular, the cortisol response in the first hour of waking is complex (Fries et al., 2009). Even small variations of the waking sampling point in our study within this window could represent different phases of the response, increasing sampling noise. Verbal report from the participants in this study and previous research of aging participants (Almeida et al., 2009; Kraemer et al., 2006) indicate we could expect generally high levels of compliance, but adherence to instructions cannot be known because the diurnal samples were taken at home. The current data do not allow us to directly quantify individuals' reactions to the testing appointment as there is no comparison salivary measure at the same time on a different weekday. However, most of the participants showed start levels either higher or very close to those predicted by their linearly modeled diurnal rhythm. This may be a conservative estimate of the neuroendocrine response to the testing appointment, given that the diurnal rhythm is nonlinear with the sharpest drop in diurnal cortisol over the first hours postwaking. We also report a range of declining, flat, and increasing reactive cortisol levels (in response to the appointment) which is at odds with the characteristic decreasing circadian rhythm. Our cross-sectional design and participants' narrow age-range preclude direct comment on causal relationships between cortisol and brain structure in aging but rather give an indication of their associations at this specific time in older age. Although the data partially corroborate previous work, a prospective design is necessary to fully address these issues. The self-selecting group of males studied had relatively good health, meaning that variance in age-related brain changes and cortisol levels may be comparatively modest. It is possible that our results are a conservative estimate of the relationship between cortisol and WM characteristics in the aging population and apply only to males. Given gender differences in both GC secretion (Otte et al., 2005) and its relation to cognitive function in older age (Beluche et al., 2010), attention could also be turned to exploring GC-WM relationships in an equivalent female sample. Finally, given that the effects sizes reported above are around a magnitude of 0.2, larger studies with greater statistical power would be required to more reliably detect the effects reported here.

These data therefore provide a useful starting point from which previously under-explored associations between specific WM tract measures and both diurnal and reactive cortisol in normal healthy older humans can be studied in future work, and may also be relevant to investigations of the effects of everyday stress and other conditions whose hallmarks include altered HPA axis functioning, such as depression. Nevertheless, the causal chain underlying these associations is unclear. Although elevated cortisol itself may affect WM structure through mechanisms discussed previously, other (non-GC) mechanisms could equally affect changes in WM with age which impair the functioning of supra-thalamic HPA axis regulation, elevating cortisol levels as a by-product. In addition, our understanding of the cognitive and functional roles of major WM tracts remains limited, precluding confident inferences about the possible cognitive sequelae of the relationships reported herein. Future research will investigate whether, and to what degree, cortisol-cognition relationships are mediated by WM integrity, or attenuated by other pertinent covariates which might also be detrimental to WM structure in older age.

## Disclosure statement

The authors declare that they have no actual or potential conflicts of interest.

## Figures and Tables

**Table 1:** Descriptive statistics of study variables

Variable	Units	n	Mean	SD
Waking	nmol/L	89	24.00	10.59
Evening	nmol/L	84	3.47	2.75
Diurnal	nmol/L	84	−20.77	9.72
Start	nmol/L	86	16.39	7.77
End	nmol/L	89	12.67	6.07
Reactive	nmol/L	86	−3.87	7.19
WMH	mm^3^	89	12,672.63	11,037.29
Genu	FA	85	0.40	0.05
	MD (×10^−6^ mm^2^/s)	85	789.18	79.14
Splenium	FA	88	0.49	0.07
	MD (×10^−6^ mm^2^/s)	88	979.63	183.73
Arcuate	FA	87	0.44	0.04
	MD (×10^−6^ mm^2^/s)	87	649.75	42.27
ATR	FA	81	0.33	0.03
	MD (×10^−6^ mm^2^/s)	84	767.36	61.58
Cingulum	FA	86	0.41	0.04
	MD (×10^−6^ mm^2^/s)	86	653.89	42.75
Uncinate	FA	85	0.33	0.03
	MD (×10^−6^ mm^2^/s)	85	769.64	58.12
ILF	FA	87	0.38	0.04
	MD (×10^−6^ mm^2^/s)	87	801.95	98.00

Waking: salivary cortisol levels taken at home on waking; evening: salivary cortisol levels taken at home at around 10 PM; diurnal: the slope (b-a) between waking and evening; start: salivary cortisol levels taken at the start of a cognitive testing appointment; end: salivary cortisol levels taken at the end of a cognitive testing appointment; reactive: the slope (b-a) between start and end. Untransformed measures are reported. Key: ATR, anterior thalamic radiation; FA, fractional anisotropy; ILF, inferior longitudinal fasciculus; MD, mean diffusivity; WMH, white matter hyperintensity volume (mm^3^).

**Table 2:** Correlations between cortisol levels and general white matter indices

	Waking	Evening^a^	Diurnal	Start^a^	End^a^	Reactive
gFA	0.04	−0.13	−0.02	−0.07	−0.06	0.09
gMD	0.07	0.23^b^	−0.06	0.25^c^	−0.11	−0.39^d^
WMHs^e^	0.04	0.04	0.01	0.24^c^	0.21^c^	−0.11

Key: gFA, general factor of tract fractional anisotropy; gMD, general factor of tract mean diffusivity; WMHs, white matter hyperintensity volume (mm^3^).

a Log transformed.

b Trend (*p* < 0.08).

c *p* < 0.05.

d *p* < 0.001.

e Square root transformed.

**Table 3:** Correlations between cortisol levels and white matter tract fractional anisotropy (top) and mean diffusivity (bottom)

	Waking	Evening^a^	Diurnal	Start^a^	End^a^	Reactive
Genu	0.07	−0.10	−0.06	−0.04	−0.03	0.04
Splenium	0.15	0.02	−0.22^c^	−0.04	−0.13	−0.05
Arcuate	0.02	−0.11	−0.02	0.04	0.00	0.00
ATR	−0.01	−0.04	−0.02	−0.03	−0.15	−0.03
Cingulum	−0.08	0.03	0.04	−0.09	−0.07	0.08
Uncinate	0.06	−0.03	−0.01	−0.08	−0.03	0.13
ILF	0.06	−0.18	−0.10	−0.11	−0.04	0.14
Genu	−0.00	0.07	0.02	0.12	−0.09	−0.21^b^
Splenium	−0.13	−0.05	0.18	0.02	−0.01	−0.04
Arcuate	0.14	0.23^c^	−0.08	0.13	−0.08	−0.22^c^
ATR	0.09	0.21^b^	−0.01	0.21^b^	0.05	−0.19
Cingulum	0.04	0.11	−0.02	0.16	0.01	−0.20
Uncinate	0.13	0.20	−0.13	0.24^c^	−0.05	−0.35^d^
ILF	−0.07	0.18	0.12	0.27^c^	0.02	−0.28^c^

Key: ATR, anterior thalamic radiation; ILF, inferior longitudinal fasciculus.

a Log transformed.

b Trend (*p* < 0.08).

c *p* < 0.05.

d *p* < 0.01.
