# Supplementary material for: Brain white matter integrity and cortisol in older men: the Lothian Birth Cohort 1936
Source: Neurobiol Aging. 2015 Jan;36(1):257–64. doi: 10.1016/j.neurobiolaging.2014.06.022 (PMC4274312; doi:10.1016/j.neurobiolaging.2014.06.022)
Supplement: Supplementary Material [file mmc1.docx]

**Supplementary Material**

The cognitive testing appointment took place in the Department of Psychology, University of Edinburgh. Participants provided a salivary cortisol sample on arrival (START sample) and then sat the following cognitive tests in specific order:

The Self-Ordered Pointing Task (Petrides & Milner, 1982). The SOPT was administered using a computerised version, containing a grid of 12 abstract designs (MacPherson et al., 2002) on a touchscreen interface (iiyama ProLite T2250MTS 22” 1920 x 1080). The participant was required to select each design only once, choosing an item that they have not previously selected. Testing continued until 12 selections have been made. Following each choice, the order of some of the items in the grid was rearranged to ensure participants remember the previously-chosen images by their appearance rather than their location. The test ended after three trials have been completed and the trials are self-paced.

Reversal Learning (Rolls, Hornak, Wade & McGrath, 1994). We used a modified version of a previously-reported neuroimaging paradigm (Hampton & O’Doherty, 2007). We used a deterministic contingency (given the large amount of training required for probabilistic versions) and altered the images from US cents to British pence. Participants were presented with 2 fractal images with the aim of determining which selection will allow them to make the most money. One image will always give a win of 25p, and the other always a loss of 25p. Once the correct image is correctly identified (indicated by 8 consecutive correct selections), the stimulus-reward contingency was reversed. This pattern continued for 50 trials, allowing a maximum of 5 reversals after the initial contingency has been learned.

Tower Test (D-KEFS; Delis, Kaplan & Kramer, 2001). Successful completion of the Tower test (taken from the D-KEFS) required solving 9 problems, each beginning with wooden discs in a specific configuration on a 3-peg board. The objective is to move the discs such that the participant creates a wooden tower depicted in a target image in as few moves as possible within a specified time limit (which increases as problems become more complex, up to 240s). The participant may only move one disc at a time, can never place a larger disc on top of a smaller one, and these instructions are displayed at the foot of the target stimuli.

Faux Pas Test (Stone, Baron-Cohen & Knight, 1998). This task requires participants to identify whether a protagonist said something awkward, or something they should not have said in 20 short stories (10 containing a faux pas). Participants read the stories at their own pace and were instructed to tell the experimenter when they had finished each one. They were then asked a series of questions about each story to determine whether the participant understood that a faux pas had occurred, including 2 factual control questions to ensure general understanding of the story. The story remained in front of participants at all times. The audio-taped responses were marked in accordance with scoring guidelines (<http://www2.psy.uq.edu.au/~stone/Faux_Pas_Recog_Test.pdf>).

Simon Task (Simon, 1969). We administered a version of the Simon Task reported by di Pellegrino et al., (2007), translated into English. Participants were required to respond as quickly and accurately as possible to the appearance of a red or green square on a computer screen by pressing the red or green key on the keyboard (positioned on the A and L keys respectively of a QWERTY keyboard). A single square appeared on either the left or right of the screen, making the required response for a red square incongruent if it appears on the right.

Dilemmas Task (Greene et al., 2001). The task was presented on the computer and comprised 11 high-conflict scenarios used by Koenigs and colleagues (2007) and initially Greene et al. (2001). Non-moral and low-conflict dilemmas were excluded altogether as 1) the literature suggests that only those containing hi-conflict moral content demonstrate sensitivity to frontal lobe damage and 2) many of these scenarios are not dilemmas at all (Kahane & Shackel, 2008). Progress through the task was self-paced.

Following completion of these cognitive tests, the END salivary cortisol sample was provided.

**References**

Delis, D. C., Kaplan, E., & Kramer, J. H. 2001. *Delis Kaplan Executive Function System: Technical Manual*. San Antonio, TX: The Psychological Corporation.

Greene, J. D., Sommerville, R. B., Nystrom, L. E., Darley, J. M., & Cohen, J. D. 2001. An fMRI investigation of emotional engagement in moral judgment. Science*,* 293(5537), 2105–2108.

Kahane, G., & Shackel, N. 2008. Do abnormal responses show utilitarian bias? Nature, 452, doi:10.1038/0678.

Koenigs, M., & Tranel, D. 2007. Irrational economic decision-making after ventromedial prefrontal damage: evidence from the Ultimatum Game. J. Neurosci., 27(4), 951–956.

Hampton, A.N., & O’Doherty, J.P. 2007. Decoding the neural substrates of reward-related decision making with functional MRI. PNAS, 104(4), 1377-1382

MacPherson, S. E., Phillips, L. H., & Della Sala, S. 2002. Age, executive function and social decision making: A dorsolateral prefrontal theory of cognitive aging. Psychol. Aging, 17(4), 598–609.

di Pellegrino, G., Ciaramelli, E., & Làdavas, E. 2007. The regulation of cognitive control following rostral anterior cingulate cortex lesion in humans. J. Cogn. Neurosci., 19(2), 275–286.

Petrides, M., & Milner, B. 1982. Deficits on subject-ordered tasks after frontal-and temporal-lobe lesions in man. Neuropsychologia, 20(3), 249–262.

Rolls, E.T., Hornak, J., Wade, D., & McGrath, J. 1994. Emotion-related learning in patients with social and emotional changes associated with frontal lobe damage. J. Neurol. Neurosurg. Psychiatry, 57(12), 1518–1524.

Simon, J. R. 1969. Reactions towards the source of stimulation. J. Exp. Psychol., 81, 174-176.

Stone, V. E., Baron-Cohen, S., & Knight, R. T. 1998. Frontal lobe contributions to theory of mind. J. Cogn. Neurosci., 10(5), 640–656.

Table S1. Effect of controlling WMH for ICV on correlation magnitudes with cortisol.

|  | Waking | Evening^a^ | Diurnal | Start^a^ | End^a^ | Reactive |
| --- | --- | --- | --- | --- | --- | --- |
| **WMH^b^** | .04 | .04 | .01 | **.24*** | **.21*** | -.11 |
| **WMH_ICV^b^** | .03 | .06 | .02 | **.25*** | **.21**^†^ | -.13 |

^†^*p*=.055, **p*<.05, ^a^ log transformed, ^b^ square root transformed, WMHs: white matter hyperintensity volume (mm^3^), WMH_ICV: white mater hyperintensity volume controlled for intracranial volume (mm^3^).

Table S2. Spearman correlations among untransformed variables (cortisol and general white matter indices).

|  | Waking | Evening | Diurnal | Start | End | Reactive |
| --- | --- | --- | --- | --- | --- | --- |
| ***g*FA** | -.02 | -.17 | .05 | -.00 | .02 | .00 |
| ***g*MD** | .11 | **.26*** | -.08 | .18 | -.17 | **-.34**** |
| **WMHs** | .04 | .05 | .00 | .20^†^ | .21^†^ | -.10 |

^†^trend (*p*<.08), **p*<.05, ****p*<.01, *g*FA: general factor of tract fractional anisotropy, *g*MD: general factor of tract mean diffusivity, WMHs: white matter hyperintensity volume (mm^3^).


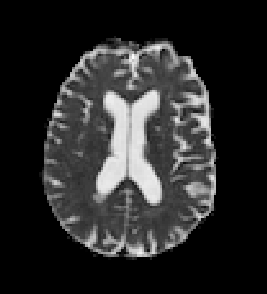

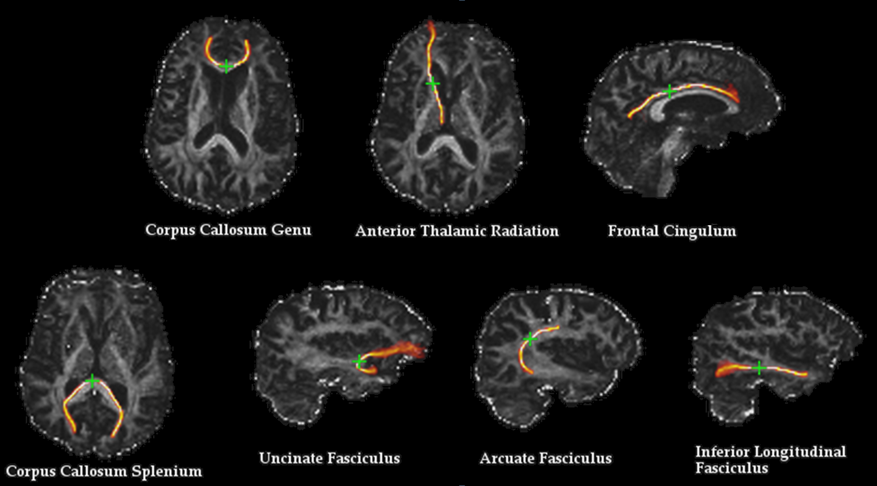


**B**

**A**

Figure S1. A: An example MD image. B: example of the tracts obtained in an LBC1936 participant using probabilistic neighbourhood tractography as implemented in the TracoR package.


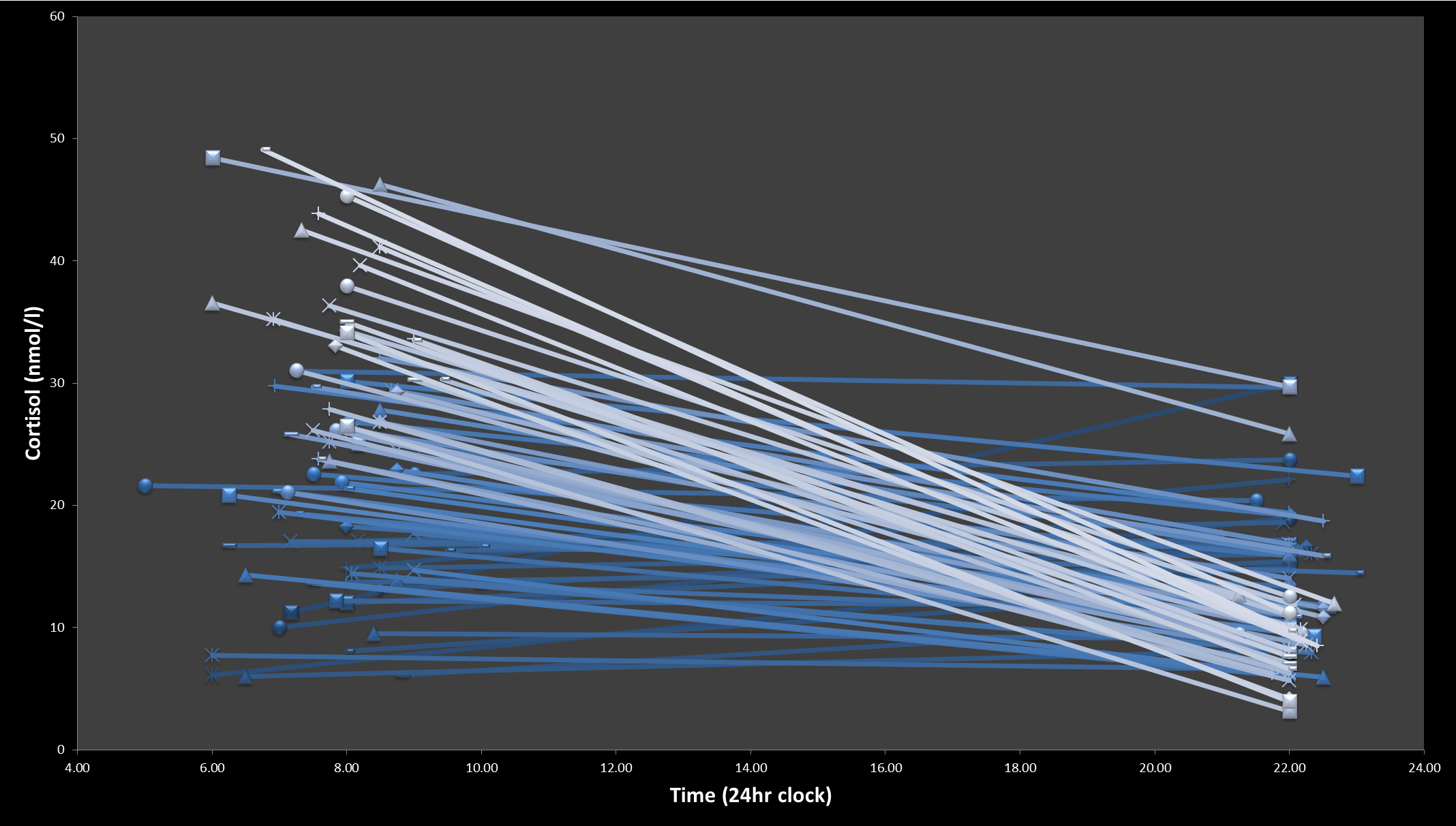


Figure S2. Individual diurnal cortisol profiles by sampling time.


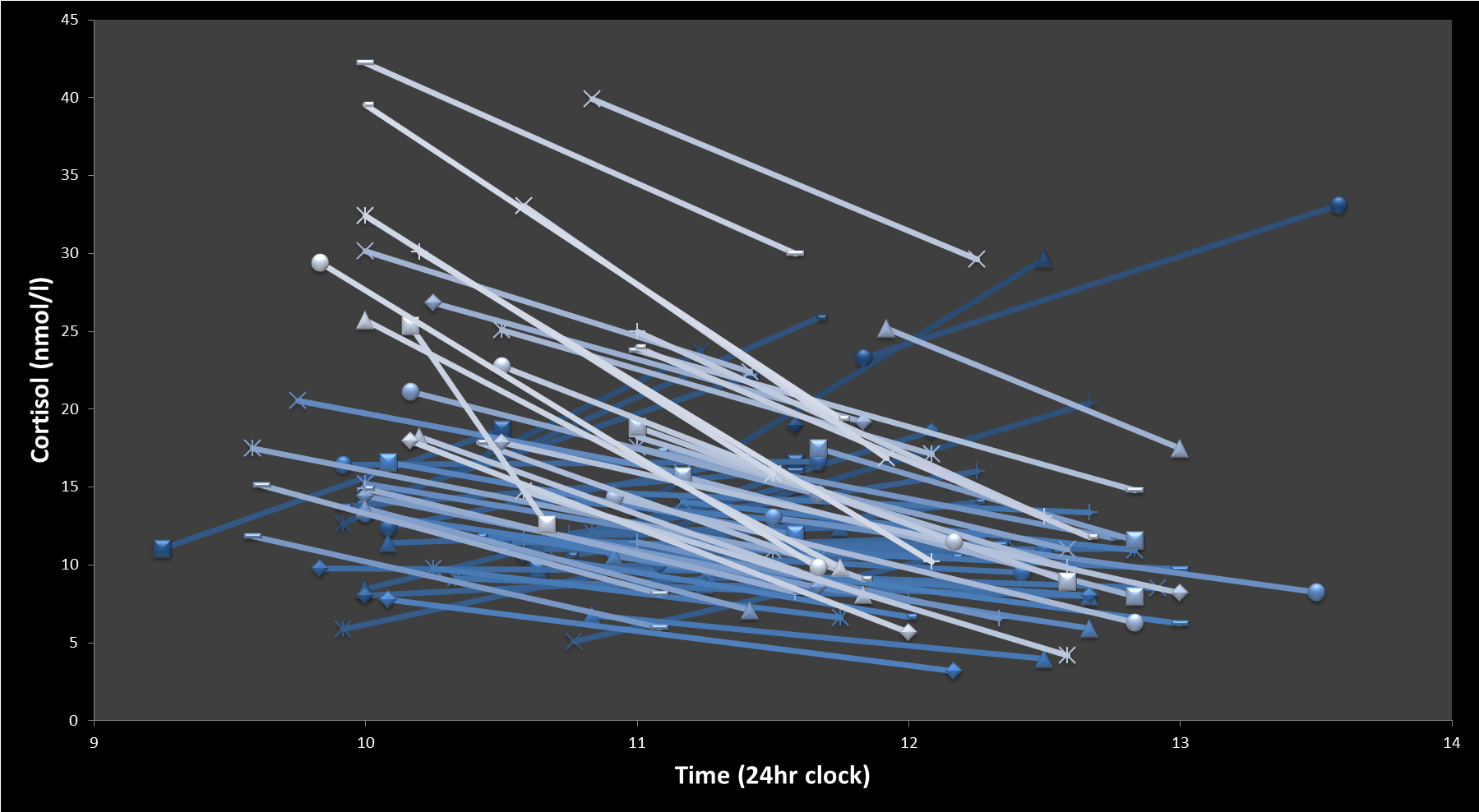


Figure S3. Individual reactive cortisol profiles by sampling time.

White Matter Hyperintensity volume (√mm^3^)

B

A

Cortisol (log nmol/l)

Figure S4. Scatter plots of the relationship of white matter hyperintensity volume to A) START and B) END cortisol levels.
